# Modeling Viral Infectious Diseases and Development of Antiviral Therapies Using Human Induced Pluripotent Stem Cell-Derived Systems

**DOI:** 10.3390/v7072800

**Published:** 2015-07-13

**Authors:** Marta Trevisan, Alessandro Sinigaglia, Giovanna Desole, Alessandro Berto, Monia Pacenti, Giorgio Palù, Luisa Barzon

**Affiliations:** 1 Department of Molecular Medicine, University of Padova, via A. Gabelli 63, 35121 Padova, Italy; E-Mails: marta.trevisan@unipd.it (M.T.); giovanna.desole@studenti.unipd.it (G.D.); alessandro.berto@studenti.unipd.it (A.B.); giorgio.palu@unipd.it (G.P.); 2 Veneto Institute of Oncology IOV IRCCS, via Gattamelata 64, 35128 Padova, Italy; E-Mail: alessandrosinigaglia@yahoo.it; 3 Microbiology and Virology Unit, Padova University Hospital, via Giustiniani 2, 35128 Padova, Italy; E-Mail: monia.pacenti@sanita.padova.it

**Keywords:** human induced pluripotent stem cells, viral infection, patient-specific disease model, genome editing, genetic susceptibility, antiviral resistance, CRISPR/Cas9, personalized therapy, hepatitis C virus, human immunodeficiency virus

## Abstract

The recent biotechnology breakthrough of cell reprogramming and generation of induced pluripotent stem cells (iPSCs), which has revolutionized the approaches to study the mechanisms of human diseases and to test new drugs, can be exploited to generate patient-specific models for the investigation of host–pathogen interactions and to develop new antimicrobial and antiviral therapies. Applications of iPSC technology to the study of viral infections in humans have included *in vitro* modeling of viral infections of neural, liver, and cardiac cells; modeling of human genetic susceptibility to severe viral infectious diseases, such as encephalitis and severe influenza; genetic engineering and genome editing of patient-specific iPSC-derived cells to confer antiviral resistance.

## 1. Introduction

Every year, infectious diseases kill millions of people worldwide. The recent human outbreak of Ebola virus infection in West Africa has dramatically shown the tremendous effects of a highly lethal pathogen, against which no effective drugs or vaccines are available [1]. Other infectious diseases, such as malaria, tuberculosis, AIDS, influenza, continue to be common causes of death especially in low income countries and represent global health emergencies that require strengthened interventions. At present, the only successful strategies to treat infectious diseases are based on targeting the infectious agent, but this approach becomes ineffective with the relentless emergence of antidrug resistance.

The recent advances in the knowledge of infectious disease pathogenesis and host factors involved in susceptibility or resistance to infectious agents have opened new perspectives for the development of personalized therapeutic interventions based on enhancing host immune response and on conferring a resistance state to a susceptible individual. The latter approach can be pursued by improving our understanding of the molecular and genetic basis of human susceptibility or resistance to infectious diseases and by developing patient-specific models for the investigation of infectious diseases and to test new therapies.

So far, information on host factors that restrict viral infection has been achieved from large genome-wide association studies and through the application of large-scale ectopic expression and gene silencing screens to identify sets of genes that control viral replication *in vitro* or that are required by specific pathogens to survive and replicate in the host cell [2]. In addition, *in vitro* tests on peripheral blood cells from patients with different infectious disease phenotypes have led to the identification of some genes involved in innate immune response as significantly associated with an increased risk of severe disease [3]. This approach is, however, not feasible when the disease phenotype is restricted to some tissues or cells, which are not accessible *in vivo*, such as the brain in viral encephalitis or the liver in viral hepatitis.

The recent biotechnology breakthrough on cell reprogramming and generation of induced pluripotent stem cells (iPSCs) [4], which has revolutionized the approaches to study the mechanisms of genetic and degenerative diseases and to test new drugs, can be exploited to generate patient-specific models for the investigation of host-pathogen interactions. Patient-specific iPSC-derived systems represent versatile, non-invasive, ethically sustainable, and cruelty-free platforms to analyze the innate immune response to pathogens and to test new therapies. Here we will review the applications of iPSCs to the study of viral infections in humans that have been recently reported in the literature, which show the potentialities of this new biotechnological platform. These studies include *in vitro* modeling of viral infections of neural, liver, and cardiac cells; modeling of human genetic susceptibility to severe viral infectious diseases, such as encephalitis and severe influenza; genetic engineering and genome editing of patient-specific iPSC-derived cells to confer antiviral resistance, with applications for the development of therapies against human immunodeficiency virus (HIV) and hepatitis virus infection.

## 2. Induced Pluripotent Stem Cell-Derived Models of Diseases

The advent of the reprogramming technology that allows generating patient-specific iPSCs from differentiated somatic cells of the body has provided unprecedented human models to study both disease pathology in different genetic backgrounds and their response to therapy. Actually, human iPSCs have been generated from a variety of somatic cells, e.g., fibroblasts, keratinocytes, peripheral blood cells, and have been differentiated into almost any cell type of the body, including disease-relevant cell types, like cardiomyocytes, hepatocytes, and neurons [5]. If derived from patients with a disease phenotype, these cells will express the entire genetic background of the patient, including not only known gene mutations, if present, but also all of the genetic modifiers that have important, yet unknown, roles in disease pathogenesis [5].

### 2.1. Generation of iPSCs

**Figure 1:**
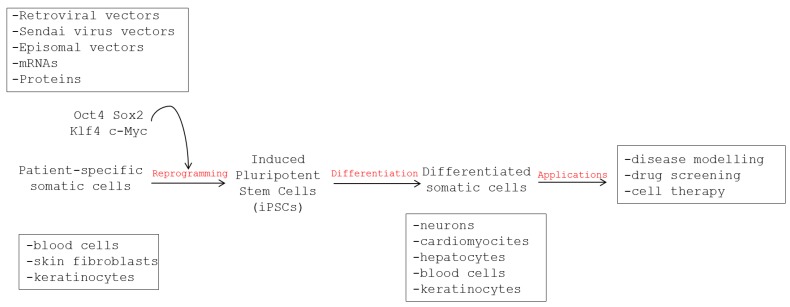
Representation of the workflow for the derivation of patient-specific induced pluripotent stem cells, their differentiation in somatic cells and tissues, and their use for disease modeling, drug screening, and development of personalized therapies.

The generation of iPSCs was first achieved in 2006 by Takahashi and Yamanaka [4], who demonstrated that cells with embryonic stem cell features could be derived from mouse fibroblasts by ectopic expression of four stem cell transcription factors (*i.e*., Oct4, Sox2, Klf4, and c-Myc). In the following year, iPSCs were generated for the first time from human somatic skin cells by using similar reprogramming protocols [6,7]. Like embryonic stem cells, human iPSCs can be grown indefinitely and differentiated into a variety of cells, but without the use of embryos or somatic cell nuclear transfer (Figure 1).

So far, different methods have been set up for the generation of iPSCs and efficient and standardized reprogramming protocols are now available [8]. The first methods exploited integrating retroviral vectors for the delivery of reprogramming factors, which, however, have been associated with the risk of tumor development due to insertional mutagenesis and/or transgene reactivation. Efficient and safe non-integrating methods based on the use of Sendai viral vectors, episome plasmid vectors, synthetic mRNAs and small molecules have been subsequently developed and are now widely applied in stem cell laboratories [8]. Particular attention is currently also given to the substrates used to generate human iPSCs in order to obtain GMP-grade cells usable for clinical purposes. The majority of reprogramming protocols are carried out, in fact, by using a layer of mitotically inactivated mouse embryonic fibroblasts (MEFs), which pose the risk of xeno-factor contamination. The usage of MEFs may, in fact, transfer exogenous antigens, unknown viruses, or zoonotic pathogens to iPSCs, contaminating the culture, as it was already shown for immunogenic non-human sialic acid N-glycolylneuraminic acid (Neu5Gc), detected on the surface of human embryonic stem cells maintained on MEF feeder [9]. This contamination might represent a problem in the setup of infectious disease models and especially in the development of iPSC-based therapies. In order to overcome this issue, many research groups have been working on generating human iPSCs using human cells as feeder layers [10,11] or even, more recently, using the same iPSCs lines to generate, by differentiation, fibroblast-like cells to be employed as feeder layer [12]. Extracellular matrix- or synthetic-based substrates such as Matrigel^®^ or vitronectin have also been extensively studied to generate feeder-free human iPSCs. For these latter methods, however, the reprogramming efficiencies are lower [13,14].

### 2.2. Features of iPSCs

The biological characteristics of human iPSCs are very similar to those of embryonic stem cells (*i.e*., pluripotent cells derived from preimplantation stage embryos). Like embryonic stem cells, iPSCs have unlimited self-renewal capacity and the potential to differentiate into all somatic cell types of the body. Different tests are used to define the pluripotent stem cell features of iPSCs, including analysis of expression of stem cell marker genes (such as alkaline phosphatase, Nanog, Oct4), whole gene expression profiling, promoter methylation analysis, and evaluation of the ability of iPSCs to differentiate into cells of the three germ cell layers, *i.e.*, ectoderm, mesoderm, and endoderm, by induction of teratomes *in vivo* or by the Embryoid bodies (EBs) test *in vitro*.

Several protocols are available for the differentiation of iPSCs into a variety of cell types, including neurons, cardiomyocytes, hepatocytes, keratinocytes, and hematopoietic cells [15]. There are three general approaches to initiate differentiation: the first method is based on the fact that *in vitro* differentiation recapitulates the stepwise stages of embryological development and exploits the formation of EBs, *i.e.*, 3D cellular aggregates obtained by withdrawal of undifferentiating stimuli such as adhesion and b-FGF, and the subsequent spontaneous trigger of the differentiation pathway [16,17]. In the second approach, pluripotent stem cells are grown directly on stromal cells, and differentiation takes place in contact with these cells [18]. The third protocol starts with cells growing in a uniform monolayer on extracellular matrix proteins [19,20]. All these different approaches offer advantages and disadvantages, but share the delivery in cell growth medium of key factors that regulate the events of cell differentiation during embryonic development. Since the resulting differentiated cells are heterogeneous and may maintain immature characteristics, selection of specific cell subtypes by cell sorting and antibiotic selection is needed for applications in disease modeling and regenerative medicine. For these applications, cells can be grown in co-cultures and in three-dimensional scaffolds [21] to generate tissues, such as liver [22], cardiac tissue [23], and neural tissue [24].

### 2.3. Disease Modeling Using Human iPSCs

Human iPSCs have been applied to the modeling of a variety of human diseases [25,26]. Most applications regarded pathogenesis studies, drug testing, and drug discovery for genetic and degenerative diseases, and, among these, mainly cardiovascular and neurological diseases [5], while applications in infectious diseases have been very limited (Figure 1). For example, several studies used patient-specific iPSC-derived cardiomyocytes to model long QT syndrome and other arrhythmia syndromes and used the cells to rescue the disease phenotype and as platform for drug testing *in vitro* [27,28,29,30]. Likewise, *in vitro* models of monogenic and multi-factorial neurological and metabolic diseases have been set up using patient-specific iPSC-derived cells [31,32,33,34,35,36,37].

The development of *in vitro* models of human diseases based on patient-specific iPSC-derived cells requires standardized and reproducible methods of reprogramming and cell differentiation, in order to minimize technical variability and biases. In addition, the setup of robust and simple assays for the detection of specific disease traits is required to analyze the disease phenotype in patient-derived cells (e.g., measurement of amyloid-β and phospho-tau in neural cell lysates as a marker of Alzheimer’s disease [35]; electrophysiology measurements to analyze alterations in ion channels [27]). These assays should be suitable for scaling up, especially if the iPSC-derived cell platforms are used for high-throughput drug screening or toxicity studies. To this aim, automated cell cultures and lab-on-chip platforms may be used for high throughput analyses [38,39], including the modeling of viral infections *in vitro* [40,41]. Adequate controls are also required to distinguish disease-specific phenotypes from inter-individual variability or technical variability related to iPSCs generation. Controls for monogenic disease models may be obtained by rescuing the mutated gene in iPSCs by targeted gene correction. Gene correction can now be efficiently achieved through homologous recombination using zinc-finger nucleases (ZFNs), transcription activator-like effector nucleases (TALENs), or CRISPR/Cas9 nucleases, *i.e.*, a gene editing system composed of clustered regularly interspaced short palindromic repeats (CRISPRs) together with CRISPR-associated nuclease (Cas) [42]. These techniques can also be used to introduce specific mutations in iPSCs to generate disease-specific genotypes. However, these approaches are quite labor intensive and can be applied only to diseases associated with known mutations. As an alternative, well-characterized iPSCs derived from matched healthy individuals can be used as controls.

## 3. Use of Human iPSCs to Model Viral Infections

The use of human iPSCs is particularly helpful to set up *in vitro* cultures of normal human cells for viruses that are strictly species-specific or that can grow only in a limited set of human cell types, like herpes simplex virus (HSV) and varicella zoster virus (VZV), which have tropism for neural cells and establish latency in sensory neurons; human cytomegalovirus (HCMV), which can be isolated and propagated in human endothelial cells; hepatitis B (HBV) and hepatitis C (HCV) viruses, which can be grown in hepatocytes. The availability of human iPSC-derived differentiated cells allows setting up potentially unlimited and easy to handle cell systems for the investigation of viral tropism, pathogenesis, latency, reactivation, and interaction with the human host. Applications of human iPSCs to model viral infections and relevant findings reported in the literature are summarized in Table 1.

### 3.1. Human Cytomegalovirus Infection

Human cytomegalovirus is a betaherpesvirus that has tropism for vascular endothelial cells, blood monocytes, and neural cells [43]. Cellular determinants that regulate HCMV replication within these cell lineages are dictated largely by the stages of cell differentiation. Infection of monocytes and their progenitors favors latent HCMV infection [44,45], while differentiation of monocytes into tissue macrophages promotes viral replication [46]. Replication in endothelial cells is important for HCMV hematogenous dissemination during acute infection and for vertical transmission from mother to fetus [47,48,49,50]. Endothelial cells have also been suggested to be sites of viral persistence during latency [47]. HCMV can also infect the central nervous system (CNS) and is the most common cause of congenital CNS infection [51].

Clinical isolates of HCMV display variable tropism *in vitro* and this variability may be associated with virulence traits. Actually, laboratory adapted HCMV strains are typically grown in fibroblasts and this adaptation is associated with genetic mutations and loss of natural tropism features [48,52,53]. Limited access to normal human cells and tissues and technical difficulties in sustaining primary human cell cultures have represented an obstacle to the investigation of the mechanisms of HCMV pathogenesis and host antiviral response. To overcome these problems, human embryonic stem cells and, more recently, iPSCs have been applied to generate human neural cells and tissues and used as models to investigate HCMV infection of human neural fetal brain and cellular response *in vitro* [54,55,56]. These studies, which showed that the *in vitro* models may represent valuable surrogates of *in vivo* infection experiments [54], demonstrated that primitive neural stem cells (NSCs), similar to those present in the developing neural tube, did not support HCMV replication but instead allowed persistent infection, while lytic infection was induced upon differentiation of progenitor NSCs into neurons [55]. These results are in contrast to the results obtained in human iPSC-derived NSCs, neural progenitor cells (NPCs) and neurons, which showed that both iPSCs and iPSC-derived neurons were not permissive, while NPCs were fully permissive for HCMV replication [56].

### 3.2. Herpes Simplex Virus and Varicella Zoster Virus Infection of Neural Cells

HSV and VZV are human neurotropic alphaherpesviruses with strict tropism for the human host. Following primary infection, both viruses establish latency in sensory neurons of the dorsal root and trigeminal ganglia. Human iPSCs have been differentiated into NPCs and sensory neurons and used to model HSV and VZV infection and disease [57,58]. *In vitro* experiments showed that iPSCs supported HSV but not VZV infection, while NPCs and differentiated cell cultures containing sensory neurons could be productively infected by both viruses, which caused a significant cytopathic effect (CPE) [57].

### 3.3. Hepatitis B Virus Infection

HBV is a small DNA virus of the *Hepadnaviridae* family that selectively infects hepatocytes in the human liver. Approximately 400 million people worldwide have chronic HBV infection, of whom about one-third will develop end-stage liver disease and liver cancer [59]. Research on HBV biology and antiviral therapy has been hampered by the lack of suitable liver models of HBV infection that recapitulate HBV life cycle and its interaction with the host. Different models of HBV infection have been developed.

Human primary adult hepatocyte cultures, which are considered the gold standard, can support HBV replication *in vitro*, but these cells are difficult to maintain [60]. Co-culture of primary hepatocytes with supportive stromal cells has allowed prolonging hepatocyte viability and their differentiated phenotype [61,62].

More recently, with the identification of the bile acid pump sodium taurocholate cotrasporting polypeptide (NTCP) as a receptor for both HBV and hepatitis D virus [63], infection models have been set up by ectopic expression of NTCP in hepatocellular carcinoma cells [64] and in transgenic mice [65]. However, hepatocellular carcinoma cells have altered innate immune response and cell proliferation mechanisms, thus limiting the usefulness of this model to the study of HBV infection.

Human iPSCs have been also exploited to generate a model of HBV infection [62]. In particular, human iPSCs have been differentiated into hepatocyte-like cells and grown in co-cultures with stromal fibroblasts. Infection of cells with HBV showed that fully differentiated hepatocyte-like cells, but not cells at earlier stages of differentiation, supported productive infection. Treatment of cells with a broad-spectrum Janus kinase inhibitor to block the induction of interferon-stimulated genes allowed to maintain HBV replication over a period of three weeks, while treatment with interferon or with the antiviral drug entecavir decreased viral titer [62]. These data indicate that this platform can be used to test anti-HBV drugs, besides modeling virus–host interactions.

### 3.4. Hepatitis C Virus Infection

HCV infection is an important cause of liver cirrhosis and hepatocellular carcinoma worldwide [66]. Research on HCV infection has been hampered by the lack of relevant *in vitro* and *in vivo* models.

The most used cell culture model of HCV infection is based on the human hepatocellular carcinoma cell line Huh7 and the JFH1 replicon of HCV of genotype 2a [67]. Huh7 cells transfected with the replicon produce infectious progeny viruses and recapitulate the entire viral life cycle [68,69,70,71]. The virus produced by this system can be used to infect primary human hepatocytes, which are the natural targets for HCV and are permissive to infection both *in vitro* [72,73,74] and *in vivo* after engraftment [75,76]. Primary human hepatocytes are obtained from patient biopsies and represent a physiological model of HCV infection. They can be infected with cell culture-derived HCV JFH1 but also with HCV-positive sera. However, their use is hampered by their limited availability, variable quality, high cost, and short-term use.

In this context, hepatocyte-like cells derived from human iPSCs could represent a valuable alternative to primary hepatocytes to model HCV infection *in vitro* [77,78]. *In vitro* differentiated human hepatocytes and hepatic progenitor cells can be persistently infected with HCV and secrete infectious viral particles into the culture medium, while iPSCs are not permissive for HCV infection. Permissiveness to infection correlated with induction of the liver-specific microRNA-122 and modulation of cellular factors that affect HCV replication, such as epidermal growth factor receptor (EGFR), ephrin receptor A2 (EphA2), and phosphatidylinositol 4-kinase type III alpha (PI4KIIIa) [77]. Human liver-like cells derived from patient-specific iPSCs have been also used to generate an *in vivo* model, by their engraftment into the liver of transgenic mice [79,80,81]. These cells underwent further maturation *in vivo* and could be persistently infected by HCV for more than three months [80].

A HCV replicon system in human iPSC-derived hepatocyte-like cells has been used as an *in vitro* platform to investigate the hepatotoxicity of new combinations of drugs against HCV [82]. By using patient-specific iPSC-derived cells, this system could be applied to the analysis of drug toxicity for individual patients.

### 3.5. Enterovirus Myocarditis

Group B coxsackieviruses (RNA viruses belonging to the Enterovirus genus) are common causes of viral myocarditis [83]. These viruses enter cardiomyocytes via the coxsackievirus and adenovirus receptor (CAR), a transmembrane cell adhesion protein, which is highly expressed in cardiomyocytes, and efficiently replicate and induce necrosis in infected cells [84].

Human iPSC-derived cardiomyocytes have been used to model myocarditis induced by coxsackievirus infection [85]. These studies have shown that cardiomyocytes derived from human iPSCs express high levels of CAR and are susceptible to coxsackievirus infection. Human iPSC-derived cardiomyocytes were also infected with an engineered coxsackievirus B3 strain expressing a luciferase reporter gene and used as a platform to measure viral replication. This model allowed testing the efficacy of antiviral drugs in reducing coxsackievirus replication in human cardiomyocytes [85].

**Table 1:** Human induced pluripotent stem cell (iPSC)-derived models of viral infections.

Disease Model	Virus	iPSC-Derived Cells	Findings	Refs
Encephalitis	HCMV	Neural stem cells, neural progenitor cells, neurons	Neural progenitor cells, but not neurons, are permissive for lytic HCMV replication	[56]
Encephalitis	HCMV	Neural stem cells, neural progenitor cells, neurons	Neural stem cells allow persistent HCMV infection; neurons are permissive for lytic replication	[55]
Encephalitis	HSV, VZV	Neural progenitor cells, sensory neurons	Neural progenitor cells and sensory neurons are permissive to productive HSV and VZV infection	[57]
Hepatitis	HBV	Hepatic progenitor cells, differentiated hepatocytes	Fully differentiated hepatocyte-like cells support productive HBV infection	[62]
Hepatitis	HCV	Hepatic progenitor cells, differentiated hepatocytes	Hepatic progenitor cells and differentiated hepatocytes are permissive for HCV infection	[77,78]
Hepatitis	HCV	Hepatic progenitor cells, differentiated hepatocytes	Liver-like cells can be engrafted in the liver of transgenic mice and persistently infected by HCV	[79,80,81]
Myocarditis	Coxsackievirus	Cardiomyocytes	Cardiomyocytes are susceptible to coxsackievirus infection	[85]

## 4. Modeling Human Susceptibility to Viral Infectious Diseases Using iPSC-derived Systems

Genome wide association studies and studies of monogenic diseases have identified some polymorphisms and mutations in genes involved in innate and adaptive antiviral-immunity or encoding viral receptors as associated with increased susceptibility or resistance to specific infectious disease phenotypes and with response to antiviral therapy [2,86]. For example, mutations of the C-C chemokine receptor type 5 gene (*CCR5*) confer resistance to HIV-1 infection, mutations of the Toll-like receptor 3 (*TLR3*) and *UNC93B1* genes are associated with susceptibility to HSV-1 encephalitis, a polymorphism upstream of interleukin 28B (*IL28B*) is associated with HCV spontaneous clearance and response to treatment with IFN-α [2]. Based on the clinical observation of the high variability of infectious disease phenotypes in different individuals, it is conceivable that host genetic background plays an important role in the outcome of many infections and that the spectrum of genetic polymorphisms and mutations associated with viral disease phenotypes is larger than currently known. Patient-specific iPSCs can be used to derive relevant cell types to model viral infectious disease phenotypes and virus-host interactions, in order to get clues on the genetic basis and the mechanisms of individual susceptibility (or resistance) to severe disease. Applications of iPSCs to model genetic susceptibility to viral infectious diseases have been reported so far for HSV encephalitis and severe influenza, as described below and summarized in Table 2.

### 4.1. Herpes Simplex Virus Encephalitis

Investigation of children with genetic predisposition to develop HSV-1 encephalitis was one of the first applications of iPSCs to the study of patient-specific susceptibility to infectious diseases. Childhood herpes simplex encephalitis is a rare, life threatening condition that predisposes otherwise healthy children to encephalitis during primary HSV-1 infection. This syndrome has been associated with mutations in the *TLR3* gene, a key factor for natural immunity to HSV-1 in the CNS, or with mutations in genes encoding factors involved in TLR3 response, such as *UNC93B1* [87,88,89,90].

By using the iPSC technology, NSCs, neurons, astrocytes, and oligodendrocytes were derived from TLR3- and UNC-93B-deficient patients and from control subjects and tested *in vitro* [91]. Experiments showed that neurons and oligodendrocytes from TLR3- and UNC-93B-deficient patients had an impaired IFN-β and IFN-λ1 response to the dsRNA analogue polyinosinic:polycytidylic acid (poly(I:C)) and to HSV-1. In addition, neurons and oligodendrocytes from UNC-93B-deficient patients and neurons from TLR3-deficient patients were much more susceptible to HSV-1 infection than control cells [91]. Impaired interferon response and HSV-1 susceptibility phenotype could be rescued by transient expression of the corresponding wild-type allele and by treatment with exogenous IFN-α or IFN-β but not IFN-λ1, thus indicating that impaired IFN-α/β intrinsic immunity to HSV-1 in the CNS may underlie the disease pathogenesis [91].

### 4.2. Severe Influenza

Influenza virus infection typically causes a self-limiting respiratory disease, but can occasionally cause life-threatening acute respiratory distress syndrome (ARDS). The presence of co-morbidities is a risk factor for ARDS, but in some cases this condition may occur in otherwise healthy individuals. A recent study demonstrated that severe influenza may result from a single-gene inborn error of immunity [92]. In this study, two compound heterozygous mutations in the transcription factor interferon regulatory factor 7 (*IRF7*) gene were identified by whole-exome sequencing in an otherwise healthy seven year-old girl who suffered life-threatening ARDS during primary infection with pandemic H1N1 2009 influenza virus. Interferon regulatory factor 7 is a transcription factor that amplifies type I and type III interferon responses to viral infection. *In vitro* functional assays demonstrated that the two mutations caused loss-of-function of IRF7. Patient’s peripheral blood mononuclear cells (PBMCs) and plasmacytoid dendritic cells infected with influenza A virus showed a significant down-regulation of type I and type III interferon genes in comparison with healthy donors and deficient IFN-α2 production after infection with 11 different viruses or stimulation with TLR agonists. To model the impact of the *IRF7* mutations on patient’s respiratory tract, pulmonary epithelial cells were generated from patient’s iPSCs. These cells produced reduced amounts of type I IFN and displayed increased influenza virus replication in comparison with control cells. Treatment with IFN-α2b, IFN-β, or IFN-λ1 rescued this phenotype. These findings suggested that impairment of IRF7-dependent amplification of IFN response in dendritic cells and in the pulmonary epithelium may have contributed to patient’s ARDS [92].

**Table 2:** Human induced pluripotent stem cell (iPSC)-derived models of genetic susceptibility to viral infectious diseases.

Disease	Genetic Defect	Disease Traits in Patients	Phenotype in Human iPSC-Derived Cells	Rescue and Drug Testing	Refs
HSV encephalitis	Inactivating mutations of *TLR3* and *UNC93B1*	Predisposition to develop encephalitis during primary HSV-1 infection	Impaired IFN response to HSV infection and increased HSV replication in patient-specific neurons and oligodendrocytes	Gene addition; interferon	[91]
Severe influenza	Inactivating mutations of *IRF7*	Development of acute respiratory distress syndrome during influenza virus infection	Impaired IFN response and increased influenza virus replication in patient-specific pulmonary epithelial cells	Interferon	[92]

## 5. Human iPSC-Based Antiviral Strategies

Genetic engineering and gene editing technologies may be used to confer antiviral resistance to patient-specific cells. This result can be achieved by knocking out viral receptor genes or host co-factors that are critical for viral replication, by enhancing cell-based immunotherapy, or by targeting the viral genome. Proof-of-concept studies of these new antiviral strategies have been reported for HIV-1, HBV, and HCV, as described below and summarized in Table 3.

### 5.1. Human Immunodeficiency Virus

Human immunodeficiency virus infects immune cells and integrates into the host genome to establish its latent infection. This represents a major challenge to the development of efficient therapies and vaccines capable to cure or prevent HIV infection.

By exploiting iPSC technology in combination with gene silencing or gene editing methods, new therapeutic strategies aiming at conferring resistance to HIV in patient-specific cells have been proposed. These strategies included the use of short hairpin RNA (shRNA) or engineered nucleases, *i.e.*, ZFNs, TALENs, or the CRISPR/Cas9 system, to disrupt viral RNA or latently integrated viral genome; to engineer iPSC-derived NK cells to enhance killing of HIV-infected cells; to down-regulate or inactivate CCR5, the main co-receptor for HIV.

While the delivery of shRNAs generally can only produce a partial and transient inhibition of targeted RNA expression, the use of engineered nucleases allows to completely knocking down target sequenced by editing the cellular genome.

A highly effective engineered nuclease-based gene editing system exploits CRISPR/Cas, which has evolved in bacteria and archaea as an antiviral defense system. The antiviral activity of this system is based on the recognition of specific sequences in the target viral genome and their cleavage with the generation of site-specific DNA double-stranded breaks (DSBs) [93]. One of these systems, the type II CRISPR/Cas9 system from *Streptococcus pyogenes* [94] has been adapted to mammalian cells to generate site-specific insertions/deletions by introducing DSBs followed by non-homologous end-joining repair or to generate genome editing by introducing DSBs followed by homologous direct repair [95,96]. Likewise, the nucleases ZFNs and TALENs can be engineered and targeted to host genome for gene editing through non-homologous end-joining repair or homologous direct repair [97].

In the first anti-HIV approach that exploits iPSCs, gene-editing tools were used to excide the integrated HIV-1 provirus from the host cell genome [98,99,100] and to directly target and disrupt the reverse-transcribed HIV genome within host cells [101]. In particular, the study by Liao *et al.* [101] demonstrated that transient transfection of the CRISPR/Cas9 system in cells transduced with a lentivirus carrying an EGFP reporter led to targeted disruption of both pre-integration viral genomes and integrated proviruses. Improved HIV disruption was achieved by targeting the LTR regions or by targeting multiple viral genome sites. The HIV-targeted CRISPR/Cas9 system was effective also in latently infected T-cell lines and in iPSC-derived monocytes/macrophages (*i.e*., the cellular reservoirs for HIV), providing long-term resistance to HIV-1 challenge [101].

The second approach aimed to generate natural killer (NK) cells and to enhance their antiviral activity. To this aim, human embryonic stem cells and iPSCs were used as a source of NK cells and the ability of these cells to suppress HIV-1 infection was shown to utilize different mechanisms, including direct lysis of target cells, antibody-dependent cellular cytotoxicity, and production of chemokines and cytokines [102]. To enhance the antiviral activity, iPSC-derived NK cells were engineered to express a HIV chimeric receptor, combining the extracellular portion of CD4 to the CD3ζ intracellular signaling chain, and used to generate NK cells. *In vitro* and *in vivo* experiments using a humanized mouse model demonstrated that the engineered NK cells inhibited HIV replication in CD4+ T-cells more efficiently than their unmodified counterparts [103].

The third therapeutic strategy was based on the down-regulation or genetic modification of the CCR5 co-receptor for HIV. A proof of concept of this approach was demonstrated by co-transducing fibroblasts with a vector carrying CCR5 shRNA in addition to the reprogramming vectors to generate iPSCs [104]. Improved differentiation of iPSCs into hematopoietic cells for a potential therapeutic use can be achieved by using iPSCs derived from hematopoietic stem cells or human epithelial cells. In this regard, cord blood CD34+ cells have been used to generate anti-HIV iPSCs, which carried CCR5 shRNA in combination with a chimeric human/rhesus TRIM5α molecule [105], which was demonstrated to be a potent pre-integration inhibitor of HIV-1 infection [106]. These cells could be continually grown and differentiated into colony-forming hematopoietic progenitors, which subsequently developed into phenotypically and functionally normal macrophages resistant to HIV-1 infection [105]. Gene editing of CCR5 was achieved by introducing in target cells the naturally occurring 32-bp homozygous deletion in CCR5 (CCR5Δ32), which confers resistance to HIV-1 infection. By using a combination of TALENs, CRISPR/Cas9, and the piggyBac technology, this mutation was introduced into iPSCs with high efficiency. The modified iPSCs were then differentiated into monocytes/macrophages, which demonstrated their resistance to HIV-1 challenge [107]. Other cellular genes involved in the control of HIV infection, transcription, and replication could be also targeted as an antiviral strategy, such as in the case of knockdown of cyclin-dependent kinase (CDK2) expression in iPSC-derived macrophages by shRNA to inhibit HIV-1 transcription [108].

### 5.2. Hepatitis Viruses

Gene editing of target cells to confer viral resistance has been applied also to generate hepatocytes resistant to HCV [77]. Transplantation of hepatocytes resistant to HCV could be used as a potential life-saving therapy in HCV-positive liver transplant recipients, who experience a high risk of reinfection. To provide a proof-of-concept of the possibility to generate HCV-resistant differentiated human hepatocytes, cells at the pluripotency stage were transduced with lentiviral vectors carrying shRNAs directed at cyclophilin A and PI4KIIIa, which are important cofactors for HCV replication [109]. These cells were then differentiated into hepatocytes and infected with wild type HCV. In these cells, levels of viral replication were undetectable, like in mock infected control cells. This is an interesting therapeutic approach for an RNA virus characterized by high mutation and turnover rates, for which inhibiting a cellular rather than a viral target may offer the advantage of a higher genetic barrier to development of resistance.

For HBV therapy, gene editing was targeted to the viral genome. In particular, the CRISPR/Cas9 system was used to target the surface antigen (HBsAg)-encoding region of HBV and tested in human hepatoma cell lines and transgenic mice carrying the HBV genome and expressing HBsAg in serum [110]. A significant reduction of HBsAg levels was observed in cell culture supernatant *in vitro* and in mouse serum *in vivo*, HBsAg-positive cells were eliminated in liver tissue, and mutations in HBV genome were demonstrated [110]. In this study, no iPSC-derived systems were used. However, besides its *in vivo* use, this therapeutic strategy could be easily applied to generate *ex vivo* patient-specific cells resistant to HBV infection.

## 6. Conclusions

In this review article, we presented and discussed applications of the iPSC technology to develop *in vitro* models of human viral infections. Although the studies reported in the literature are very few in comparison with the numerous applications for other inherited and degenerative diseases, the results achieved in virology are remarkable and suggest that iPSC technology could be very useful for many other applications in the modeling of virus–human cell interactions, in the study of patient-specific viral disease outcome and response to antiviral therapies, and as platforms for the development of new antiviral strategies.

**Table 3:** Patients-specific induced pluripotent stem cell (iPSC)-based antiviral strategies.

Virus	Antiviral Strategy	iPSC-Derived Target Cells	Results	Refs
HIV	Disruption of integrated HIV genome	T cells, monocytes/ macrophages	HIV-targeted CRISPR/Cas9 disrupts reverse-transcribed and integrated HIV genome	[101]
HIV	Enhanced immune response	NK cells	Antiviral activity of iPSC-derived NKs; engineering a HIV chimeric CD4/CD3ζ receptor enhances NK activity against HIV	[102,103]
HIV	Viral receptor inactivation	Monocytes/ macrophages	Knockdown of CCR5 by shRNA; introduction of the CCR5Δ32 mutation by genome editing confers resistance to HIV-1 infection	[104,105,107]
HIV	Downregulation of viral cofactors by shRNAs	Monocytes/ macrophages	Inhibition of CDK2 and TRIM5α inhibits HIV-1 transcription	[105,108]
HCV	Downregulation of viral cofactors by siRNAs	Differentiated hepatocytes	Inhibition of cyclophilin A and PIaKIIIa inhibits HCV replication in hepatocytes	[109]

## References

[1] Heymann D.L., Chen L., Takemi K., Fidler D.P., Tappero J.W., Thomas M.J., Kenyon T.A., Frieden T.R., Yach D., Nishtar S. (2015). Global health security: The wider lessons from the west African Ebola virus disease epidemic. Lancet.

[2] Chapman S.J., Hill A.V. (2012). Human susceptibility to infectious disease. Nat. Rev. Genet..

[3] Lee M.N., Ye C., Villani A.C., Raj T., Li W., Eisenhaure T.M., Imboywa S.H., Chipendo P.I., Ran F.A., Slowikowski K. (2014). Common genetic variants modulate pathogen-sensing responses in human dendritic cells. Science.

[4] Takahashi K., Yamanaka S. (2006). Induction of pluripotent stem cells from mouse embryonic and adult fibroblast cultures by defined factors. Cell.

[5] Bellin M., Marchetto M.C., Gage F.H., Mummery C.L. (2012). Induced pluripotent stem cells: The new patient?. Nat. Rev. Mol. Cell Biol..

[6] Takahashi K., Tanabe K., Ohnuki M., Narita M., Ichisaka T., Tomoda K., Yamanaka S. (2007). Induction of pluripotent stem cells from adult human fibroblasts by defined factors. Cell.

[7] Yu J., Vodyanik M.A., Smuga-Otto K., Antosiewicz-Bourget J., Frane J.L., Tian S., Nie J., Jonsdottir G.A., Ruotti V., Stewart R. (2007). Induced pluripotent stem cell lines derived from human somatic cells. Science.

[8] Schlaeger T.M., Daheron L., Brickler T.R., Entwisle S., Chan K., Cianci A., DeVine A., Ettenger A., Fitzgerald K., Godfrey M. (2015). A comparison of non-integrating reprogramming methods. Nat. Biotechnol..

[9] Martin M.J., Muotri A., Gage F., Varki A. (2005). Human embryonic stem cells express an immunogenic nonhuman sialic acid. Nat. Med..

[10] Takahashi K., Narita M., Yokura M., Ichisaka T., Yamanaka S. (2009). Huma induced pluripotent stem cells on autologous feeders. PLoS ONE.

[11] Sugii S., Kida Y., Kawamura T., Suzuki J., Vassena R., Yin Y.Q., Lutz M.K., Berggren W.T., Izpisua Belmonte J.C., Evans R.M. (2010). Human and mouseadipose-derived cells support feeder-independent induction of pluripotent stem cells. Proc. Natl. Acad. Sci. USA.

[12] Du S.H., Tay J.C., Chen C., Tay F.C., Tan W.K., Li Z.D., Wang S. (2015). Human iPS cell-derived fibroblast-like cells as feeder layers for iPS cell derivation and expansion. J. Biosci. Bioeng..

[13] Kim H.T., Lee K.I., Kim D.W., Hwang D.Y. (2013). An ECM-based culture system for the generation and maintenance of xeno-free human iPS cells. Biomaterials.

[14] Groß B., Sgodda M., Rasche M., Schambach A., Göhring G., Schlegelberger B., Greber B., Linden T., Reinhardt D., Cantz T. (2013). Improved generation of patient-specific induced pluripotent stem cells using a chemically-defined and matrigel-based approach. Curr. Mol. Med..

[15] Sterneckert J.L., Reinhardt P., Schöler H.R. (2014). Investigating human disease using stem cell models. Nat. Rev. Genet..

[16] Doetschman T.C., Eistetter H., Katz M., Schmidt W., Kemler R. (1985). The *in vitro* development of blastocyst-derived embryonic stem cell lines: Formation of visceral yolk sac, blood islands and myocardium. J. Embryol. Exp. Morphol..

[17] Imamura T., Cui L., Teng R., Johkura K., Okouchi Y., Asanuma K., Ogiwara N., Sasaki K. (2004). Embryonic stem cell-derived embryoid bodies in three-dimensional culture system form hepatocyte-like cells *in vitro* and *in vivo*. Tissue Eng..

[18] Nakano T., Kodama H., Honjo T. (1994). Generation of lymphohematopoietic cells from embryonic stem cells in culture. Science.

[19] Nishikawa S., Nishikawa S., Hirashima M., Matsuyoshi N., Kodama H. (1998). Progressive lineage analysis by cell sorting and culture identifies FLK+VE-cadherin cells at a diverging point of endothelial and hemopoietic lineages. Development.

[20] Carpenter L., Carr C., Yang C.T., Stuckey D.J., Clarke K., Watt S.M. (2012). Efficient differentiation of human induced pluripotent stem cells generates cardiac cells that provide protection following myocardial infarction in the rat. Stem Cells Dev..

[21] Giobbe G.G., Zagallo M., Riello M., Serena E., Masi G., Barzon L., di Camillo B., Elvassore N. (2012). Confined 3D microenvironment regulates early differentiation in human pluripotent stem cells. Biotechnol. Bioeng..

[22] Takebe T., Sekine K., Enomura M., Koike H., Kimura M., Ogaeri T., Zhang R.R., Ueno Y., Zheng Y.W., Koike N. (2013). Vascularized and functional human liver from an iPSC-derived organ bud transplant. Nature.

[23] Dvir T., Timko B.P., Brigham M.D., Naik S.R., Karajanagi S.S., Levy O., Jin H., Parker K.K., Langer R., Kohane D.S. (2011). Nanowired three-dimensional cardiac patches. Nat. Nanotechnol..

[24] Choi S.H., Kim Y.H., Hebisch M., Sliwinski C., Lee S., D’Avanzo C., Chen H., Hooli B., Asselin C., Muffat J. (2014). A three-dimensional human neural cell culture model of Alzheimer’s disease. Nature.

[25] Park I.H., Arora N., Huo H., Maherali N., Ahfeldt T., Shimamura A., Lensch M.W., Cowan C., Hochedlinger K., Daley G.Q. (2008). Disease-specific induced pluripotent stem cells. Cell.

[26] Tiscornia G., Vivas E.L., Izpisúa Belmonte J.C. (2011). Diseases in a dish: Modeling human genetic disorders using induced pluripotent cells. Nat. Med..

[27] Moretti A., Bellin M., Welling A., Jung C.B., Lam J.T., Bott-Flügel L., Dorn T., Goedel A., Höhnke C., Hofmann F. (2010). Patient-specific induced pluripotent stem-cell models for long-QT syndrome. N. Engl. J. Med..

[28] Itzhaki I., Maizels L., Huber I., Zwi-Dantsis L., Caspi O., Winterstern A., Feldman O., Gepstein A., Arbel G., Hammerman H. (2011). Modelling the long QT syndrome with induced pluripotent stem cells. Nature.

[29] Ma D., Wei H., Lu J., Ho S., Zhang G., Sun X., Oh Y., Tan S.H., Ng M.L., Shim W. (2013). Generation of patient-specific induced pluripotent stem cell-derived cardiomyocytes as a cellular model of arrhythmogenic right ventricular cardiomyopathy. Eur. Heart J..

[30] Egashira T., Yuasa S., Suzuki T., Aizawa Y., Yamakawa H., Matsuhashi T., Ohno Y., Tohyama S., Okata S., Seki T. (2012). Disease characterization using LQTS-specific induced pluripotent stem cells. Cardiovasc. Res..

[31] Lee G., Papapetrou E.P., Kim H., Chambers S.M., Tomishima M.J., Fasano C.A., Ganat Y.M., Menon J., Shimizu F., Viale A. (2009). Modelling pathogenesis and treatment of familial dysautonomia using patient-specific iPSCs. Nature.

[32] Marchetto M.C., Carromeu C., Acab A., Yu D., Yeo G.W., Mu Y., Chen G., Gage F.H., Muotri A.R. (2010). A model for neural development and treatment of Rett syndrome using human induced pluripotent stem cells. Cell.

[33] Devine M.J., Ryten M., Vodicka P., Thomson A.J., Burdon T., Houlden H., Cavaleri F., Nagano M., Drummond N.J., Taanman J.W. (2011). Parkinson’s disease induced pluripotent stem cells with triplication of the α-synuclein locus. Nat. Commun..

[34] Brennand K.J., Simone A., Jou J., Gelboin-Burkhart C., Tran N., Sangar S., Li Y., Mu Y., Chen G., Yu D. (2011). Modelling schizophrenia using human induced pluripotent stem cells. Nature.

[35] Israel M.A., Yuan S.H., Bardy C., Reyna S.M., Mu Y., Herrera C., Hefferan M.P., van Gorp S., Nazor K.L., Boscolo F.S. (2012). Probing sporadic and familial Alzheimer’s disease using induced pluripotent stem cells. Nature.

[36] Rashid S.T., Corbineau S., Hannan N., Marciniak S.J., Miranda E., Alexander G., Huang-Doran I., Griffin J., Ahrlund-Richter L., Skepper J. (2010). Modeling inherited metabolic disorders of the liver using human induced pluripotent stem cells. J. Clin. Investig..

[37] Ghodsizadeh A., Taei A., Totonchi M., Seifinejad A., Gourabi H., Pournasr B., Aghdami N., Malekzadeh R., Almadani N., Salekdeh G.H. (2010). Generation of liver disease-specific induced pluripotent stem cells along with efficient differentiation to functional hepatocyte-like cells. Stem Cell Rev..

[38] Serena E., Cimetta E., Zatti S., Zaglia T., Zagallo M., Keller G., Elvassore N. (2012). Micro-arrayed human embryonic stem cells-derived cardiomyocytes for *in vitro* functional assay. PLoS ONE.

[39] Giobbe G.G., Michielin F., Luni C., Giulitti S., Martewicz S., Dupont S., Floreani A., Elvassore N. (2015). Functional differentiation of human pluripotent stem cells on a chip. Nat. Methods.

[40] Luni C., Michielin F., Barzon L., Calabrò V., Elvassore N. (2013). Stochastic model-assisted development of efficient low-dose viral transduction in microfluidics. Biophys. J..

[41] Cimetta E., Franzoso M., Trevisan M., Serena E., Zambon A., Giulitti S., Barzon L., Elvassore N. (2012). Microfluidic-driven viral infection on cell cultures: Theoretical and experimental study. Biomicrofluidics.

[42] Ding Q., Lee Y.K., Schaefer E.A., Peters D.T., Veres A., Kim K., Kuperwasser N., Motola D.L., Meissner T.B., Hendriks W.T. (2013). A TALEN genome-editing system for generating human stem cell-based disease models. Cell Stem Cell.

[43] Gerna G., Baldanti F., Revello M.G. (2004). Pathogenesis of human cytomegalovirus infection and cellular targets. Hum. Immunol..

[44] Goodrum F.D., Jordan C.T., High K., Shenk T. (2002). Human cytomegalovirus gene expression during infection of primary hematopoietic progenitor cells: A model for latency. Proc. Natl. Acad. Sci. USA.

[45] Hargett D., Shenk T.E. (2011). Experimental human cytomegalovirus latency in CD14+ monocytes. Proc. Natl. Acad. Sci. USA.

[46] Ibanez C.E., Schrier R., Ghazal P., Wiley C., Nelson J.A. (1991). Human cytomegalovirus productively infects primary differentiated macrophages. J. Virol..

[47] Fish K.N., Soderberg-Naucler C., Mills L.K., Stenglein S., Nelson J.A. (1998). Human cytomegalovirus persistently infects aortic endothelial cells. J. Virol..

[48] Hahn G., Revello M.G., Patrone M., Percivalle E., Campanini G., Sarasini A., Wagner M., Gallina A., Milanesi G., Koszinowski U. (2004). Human cytomegalovirus UL131-128 genes are indispensable for virus growth in endothelial cells and virus transfer to leukocytes. J. Virol..

[49] Bentz G.L., Jarquin-Pardo M., Chan G., Smith M.S., Sinzger C., Yurochko A.D. (2006). Human cytomegalovirus (HCMV) infection of endothelial cells promotes naive monocyte extravasation and transfer of productive virus to enhance hematogenous dissemination of HCMV. J. Virol..

[50] Weisblum Y., Panet A., Haimov-Kochman R., Wolf D.G. (2014). Models of vertical cytomegalovirus (CMV) transmission and pathogenesis. Semin. Immunopathol..

[51] Griffiths P., Baraniak I., Reeves M. (2015). The pathogenesis of human cytomegalovirus. J. Pathol..

[52] Cha T.A., Tom E., Kemble G.W., Duke G.M., Mocarski E.S., Spaete R.R. (1996). Human cytomegalovirus clinical isolates carry at least 19 genes not found in laboratory strains. J. Virol..

[53] Wang D., Shenk T. (2005). Human cytomegalovirus virion protein complex required for epithelial and endothelial cell tropism. Proc. Natl. Acad. Sci. USA.

[54] Cosset É., Martinez Y., Preynat-Seauve O., Lobrinus J.A., Tapparel C., Cordey S., Peterson H., Petty T.J., Colaianna M., Tieng V. (2015). Human three-dimensional engineered neural tissue reveals cellular and molecular events following cytomegalovirus infection. Biomaterials.

[55] Belzile J.P., Stark T.J., Yeo G.W., Spector D.H. (2014). Human cytomegalovirus infection of human embryonic stem cell-derived primitive neural stem cells is restricted at several steps but leads to the persistence of viral DNA. J. Virol..

[56] D’Aiuto L., di Maio R., Heath B., Raimondi G., Milosevic J., Watson A.M., Bamne M., Parks W.T., Yang L., Lin B. (2012). Human induced pluripotent stem cell-derived models to investigate human cytomegalovirus infection in neural cells. PLoS ONE.

[57] Lee K.S., Zhou W., Scott-McKean J.J., Emmerling K.L., Cai G.Y., Krah D.L., Costa A.C., Freed C.R., Levin M.J. (2012). Human sensory neurons derived from induced pluripotent stem cells support varicella-zoster virus infection. PLoS ONE.

[58] D’Aiuto L., Prasad K.M., Upton C.H., Viggiano L., Milosevic J., Raimondi G., McClain L., Chowdari K., Tischfield J., Sheldon M. (2015). Persistent infection by HSV-1 is associated with changes in functional architecture of iPSC-derived neurons and brain activation patterns underlying working memory performance. Schizophr. Bull..

[59] European Association for the Study of the Liver (2012). EASL clinical practice guidelines: Management of chronic hepatitis B virus infection. J. Hepatol..

[60] Gripon P., Diot C., Guguen-Guillouzo C. (1993). Reproducible high level infection of cultured adult human hepatocytes by hepatitis B virus: Effect of polyethylene glycol on adsorption and penetration. Virology.

[61] Khetani S.R., Bhatiam S.N. (2008). Microscale culture of human liver cells for drug development. Nat. Biotechnol..

[62] Shlomai A., Schwartz R.E., Ramanan V., Bhatta A., de Jong Y.P., Bhatia S.N., Rice C.M. (2014). Modeling host interactions with hepatitis B virus using primary and induced pluripotent stem cell-derived hepatocellular systems. Proc. Natl. Acad. Sci. USA.

[63] Yan H., Zhong G., Xu G., He W., Jing Z., Gao Z., Huang Y., Qi Y., Peng B., Wang H. (2012). Sodium taurocholate cotransporting polypeptide is a functional receptor for human hepatitis B and D virus. Elife.

[64] Ko C., Park W.J., Park S., Kim S., Windisch M.P., Ryu W.S. (2015). The FDA approved drug irbesartan inhibits HBV-infection in HepG2 cells stably expressing sodium taurocholate co-transporting polypeptide. Antivir. Ther..

[65] He W., Ren B., Mao F., Jing Z., Li Y., Liu Y., Peng B., Yan H., Qi Y., Sun Y. (2015). Hepatitis D virus infection of mice expressing human sodium taurocholate co-transporting polypeptide. PLoS Pathog..

[66] Lavanchy D. (2009). The global burden of hepatitis C. Liver Int..

[67] Kato T., Date T., Miyamoto M., Furusaka A., Tokushige K., Mizokami M., Wakita T. (2003). Efficient replication of the genotype 2a hepatitis C virus subgenomic replicon. Gastroenterology.

[68] Lindenbach B.D., Evans M.J., Syder A.J., Wölk B., Tellinghuisen T.L., Liu C.C., Maruyama T., Hynes R.O., Burton D.R., McKeating J.A. (2005). Complete replication of hepatitis C virus in cell culture. Science.

[69] Wakita T., Pietschmann T., Kato T., Date T., Miyamoto M., Zhao Z., Murthy K., Habermann A., Kräusslich H.G., Mizokami M. (2005). Production of infectious hepatitis C virus in tissue culture from a cloned viral genome. Nat. Med..

[70] Heller T., Saito S., Auerbach J., Williams T., Moreen T.R., Jazwinski A., Cruz B., Jeurkar N., Sapp R., Luo G. (2005). An *in vitro* model of hepatitis C virion production. Proc. Natl. Acad. Sci. USA.

[71] Kato T., Matsumura T., Heller T., Saito S., Sapp R.K., Murthy K., Wakita T., Liang T.J. (2007). Production of infectious hepatitis C virus of various genotypes in cell cultures. J. Virol..

[72] Ploss A., Khetani S.R., Jones C.T., Syder A.J., Trehan K., Gaysinskaya V.A., Mu K., Ritola K., Rice C.M., Bhatia S.N. (2010). Persistent hepatitis C virus infection in microscale primary human hepatocyte cultures. Proc. Natl. Acad. Sci. USA.

[73] Podevin P., Carpentier A., Pène V., Aoudjehane L., Carrière M., Zaïdi S., Hernandez C., Calle V., Méritet J.F., Scatton O. (2010). Production of infectious hepatitis C virus in primary cultures of human adult hepatocytes. Gastroenterology.

[74] Thomas E., Gonzalez V.D., Li Q., Modi A.A., Chen W., Noureddin M., Rotman Y., Liang T.J. (2012). HCV infection induces a unique hepatic innate immune response associated with robust production of type III interferons. Gastroenterology.

[75] Mercer D.F., Schiller D.E., Elliott J.F., Douglas D.N., Hao C., Rinfret A., Addison W.R., Fischer K.P., Churchill T.A., Lakey J.R. (2001). Hepatitis C virus replication in mice with chimeric human livers. Nat. Med..

[76] Tesfaye A., Stift J., Maric D., Cui Q., Dienes H.P., Feinstone S.M. (2013). Chimeric mouse model for the infection of hepatitis B and C viruses. PLoS ONE.

[77] Wu X., Robotham J.M., Lee E., Dalton S., Kneteman N.M., Gilbert D.M., Tang H. (2012). Productive hepatitis C virus infection of stem cell-derived hepatocytes reveals a critical transition to viral permissiveness during differentiation. PLoS Pathog..

[78] Schwartz R.E., Trehan K., Andrus L., Sheahan T.P., Ploss A., Duncan S.A., Rice C.M., Bhatia S.N. (2012). Modeling hepatitis C virus infection using human induced pluripotent stem cells. Proc. Natl. Acad. Sci. USA.

[79] Carpentier A., Tesfaye A., Chu V., Nimgaonkar I., Zhang F., Lee S.B., Thorgeirsson S.S., Feinstone S.M., Liang T.J. (2014). Engrafted human stem cell-derived hepatocytes establish an infectious HCV murine model. J. Clin. Investig..

[80] Weglarz T.C., Degen J.L., Sandgren E.P. (2000). Hepatocyte transplantation into diseased mouse liver. Kinetics of parenchymal repopulation and identification of the proliferative capacity of tetraploid and octaploid hepatocytes. Am. J. Pathol..

[81] Liu H., Kim Y., Sharkis S., Marchionni L., Jang Y.Y. (2011). *In vivo* liver regeneration potential of human induced pluripotent stem cells from diverse origins. Sci. Transl. Med..

[82] Moriguchi H., Chung R.T., Sato C. (2010). An identification of novel therapy for human hepatocellular carcinoma by using human induced pluripotent stem cells. Hepatology.

[83] Bowles N.E., Richardson P.J., Olsen E.G., Archard L.C. (1986). Detection of Coxsackie-B-virus-specific RNA sequences in myocardial biopsy samples from patients with myocarditis and dilated cardiomyopathy. Lancet.

[84] Muehlenbachs A., Bhatnagar J., Zaki S.R. (2015). Tissue tropism, pathology and pathogenesis of enterovirus infection. J. Pathol..

[85] Sharma A., Marceau C., Hamaguchi R., Burridge P.W., Rajarajan K., Churko J.M., Wu H., Sallam K.I., Matsa E., Sturzu A.C. (2014). Human induced pluripotent stem cell-derived cardiomyocytes as an *in vitro* model for coxsackievirus B3-induced myocarditis and antiviral drug screening platform. Circ. Res..

[86] Sancho-Shimizu V., Perez de Diego R., Jouanguy E., Zhang S.Y., Casanova J.L. (2011). Inborn errors of anti-viral interferon immunity in humans. Curr. Opin. Virol..

[87] Casrouge A., Zhang S.Y., Eidenschenk C., Jouanguy E., Puel A., Yang K., Alcais A., Picard C., Mahfoufi N., Nicolas N. (2006). Herpes simplex virus encephalitis in human UNC-93B deficiency. Science.

[88] Zhang S.Y., Jouanguy E., Ugolini S., Smahi A., Elain G., Romero P., Segal D., Sancho-Shimizu V., Lorenzo L., Puel A. (2007). TLR3 deficiency in patients with herpes simplex encephalitis. Science.

[89] Guo Y., Audry M., Ciancanelli M., Alsina L., Azevedo J., Herman M., Anguiano E., Sancho-Shimizu V., Lorenzo L., Pauwels E. (2011). Herpes simplex virus encephalitis in a patient with complete TLR3 deficiency: TLR3 is otherwise redundant in protective immunity. J. Exp. Med..

[90] Herman M., Ciancanelli M., Ou Y.H., Lorenzo L., Klaudel-Dreszler M., Pauwels E., Sancho-Shimizu V., Pérez de Diego R., Abhyankar A., Israelsson E. (2012). Heterozygous TBK1 mutations impair TLR3 immunity and underlie herpes simplex encephalitis of childhood. J. Exp. Med..

[91] Lafaille F.G., Pessach I.M., Zhang S.Y., Ciancanelli M.J., Herman M., Abhyankar A., Ying S.W., Keros S., Goldstein P.A., Mostoslavsky G. (2012). Impaired intrinsic immunity to HSV-1 in human iPSC-derived TLR3-deficient CNS cells. Nature.

[92] Ciancanelli M.J., Huang S.X., Luthra P., Garner H., Itan Y., Volpi S., Lafaille F.G., Trouillet C., Schmolke M., Albrecht R.A. (2015). Infectious disease. Life-threatening influenza and impaired interferon amplification in human *IRF7* deficiency. Science.

[93] Barrangou R., Fremaux C., Deveau H., Richards M., Boyaval P., Moineau S., Romero D.A., Horvath P. (2007). CRISPR provides acquired resistance against viruses in prokaryotes. Science.

[94] Jinek M., Chylinski K., Fonfara I., Hauer M., Doudna J.A., Charpentier E. (2012). A programmable dual-RNA-guided DNA endonuclease in adaptive bacterial immunity. Science.

[95] Cong L., Ran F.A., Cox D., Lin S., Barretto R., Habib N., Hsu P.D., Wu X., Jiang W., Marraffini L.A., Zhang F. (2013). Multiplex genome engineering using CRISPR/Cas systems. Science.

[96] Cho S.W., Kim S., Kim J.M., Kim J.S. (2013). Targeted genome engineering in human cells with the Cas9 RNA-guided endonuclease. Nat. Biotechnol..

[97] Kim H., Kim J.S. (2014). A guide to genome engineering with programmable nucleases. Nat. Rev. Genet..

[98] Qu X., Wang P., Ding D., Li L., Wang H., Ma L., Zhou X., Liu S., Lin S., Wang X. (2013). Zinc-finger-nucleases mediate specific and efficient excision of HIV-1 proviral DNA from infected and latently infected human T cells. Nucleic Acids Res..

[99] Ebina H., Misawa N., Kanemura Y., Koyanagi Y. (2013). Harnessing the CRISPR/Cas9 system to disrupt latent HIV-1 provirus. Sci. Rep..

[100] Hu W., Kaminski R., Yang F., Zhang Y., Cosentino L., Li F., Luo B., Alvarez-Carbonell D., Garcia-Mesa Y., Karn J. (2014). RNA-directed gene editing specifically eradicates latent and prevents new HIV-1 infection. Proc. Natl. Acad. Sci. USA.

[101] Liao H.K., Gu Y., Diaz A., Marlett J., Takahashi Y., Li M., Suzuki K., Xu R., Hishida T., Chang C.J. (2015). Use of the CRISPR/Cas9 system as an intracellular defense against HIV-1 infection in human cells. Nat. Commun..

[102] Ni Z., Knorr D.A., Clouser C.L., Hexum M.K., Southern P., Mansky L.M., Park I.H., Kaufman D.S. (2011). Human pluripotent stem cells produce natural killer cells that mediate anti-HIV-1 activity by utilizing diverse cellular mechanisms. J. Virol..

[103] Ni Z., Knorr D.A., Bendzick L., Allred J., Kaufman D.S. (2014). Expression of chimeric receptor CD4ζ by natural killer cells derived from human pluripotent stem cells improves *in vitro* activity but does not enhance suppression of HIV infection *in vivo*. Stem Cells.

[104] Kamata M., Liu S., Liang M., Nagaoka Y., Chen I.S. (2010). Generation of human induced pluripotent stem cells bearing an anti-HIV transgene by a lentiviral vector carrying an internal murine leukemia virus promoter. Hum. Gene Ther..

[105] Kambal A., Mitchell G., Cary W., Gruenloh W., Jung Y., Kalomoiris S., Nacey C., McGee J., Lindsey M., Fury B. (2011). Generation of HIV-1 resistant and functional macrophages from hematopoietic stem cell-derived induced pluripotent stem cells. Mol. Ther..

[106] Anderson J.S., Javien J., Nolta J.A., Bauer G. (2009). Preintegration HIV-1 inhibition by a combination lentiviral vector containing a chimeric TRIM5 α protein, a CCR5 shRNA, and a TAR decoy. Mol. Ther..

[107] Ye L., Wang J., Beyer A.I., Teque F., Cradick T.J., Qi Z., Chang J.C., Bao G., Muench M.O., Yu J. (2014). Seamless modification of wild-type induced pluripotent stem cells to the natural CCR5Δ32 mutation confers resistance to HIV infection. Proc. Natl. Acad. Sci. USA.

[108] Jerebtsova M., Kumari N., Xu M., de Melo G.B., Niu X., Jeang K.T., Nekhai S. (2012). HIV-1 resistant CDK2-knockdown macrophage-like cells generated from 293T cell-derived human induced pluripotent stem cells. Biology.

[109] Yang F., Robotham J.M., Nelson H.B., Irsigler A., Kenworthy R., Tang H. (2008). Cyclophilin A is an essential cofactor for hepatitis C virus infection and the principal mediator of cyclosporine resistance *in vitro*. J. Virol..

[110] Seeger C., Sohn J.A. (2014). Targeting hepatitis B virus with CRISPR/Cas9. Mol. Ther. Nucleic Acids.

